# The Effects of a Supramaximal Intermittent Training Program on Aerobic and Anaerobic Running Measures in Junior Male Soccer Players

**DOI:** 10.5114/jhk/170755

**Published:** 2023-10-11

**Authors:** Jaelson Gonçalves Ortiz, Ricardo Dantas de Lucas, Anderson Santiago Teixeira, Pedro Augusto Mohr, Luiz Guilherme Antonacci Guglielmo

**Affiliations:** 1 Physical Effort Laboratory, Sports Center, Federal University of Santa Catarina, Florianópolis - SC, Brazil.; 2 Research Group for Development of Football and Futsal, Sports Center, Federal University of Santa Catarina, Florianópolis - SC, Brazil.

**Keywords:** Carminatti’s test, football association, interval training, maximal oxygen uptake, running performance

## Abstract

This study investigated the effectiveness of supplementing regular preseason soccer training with a supramaximal intermittent shuttle-run training (ISRT) model prescribed from Carminatti’s Test peak speed (PS_T-CAR_) in aerobic performance-related indices and sprinting speed in male junior soccer players. Twenty-three national-level soccer players (mean ± SD; age 18.07 ± 0.9 y, body height 1.76 ± 0.65 m, body mass 71.9 ± 8.7 kg) were assigned to either an experimental group (EG; n = 13) performing ISRT + soccer training or a control group (CG; n = 10) that followed regular preseason soccer training alone. The following tests were applied before and after the eight-week training intervention: (i) incremental treadmill tests (VO_2max_ and lactate minimum speed – LMS); (ii) linear 30-m sprint test and Carminatti’s Test (PS_T-CAR_). Results indicated larger gains for the EG in LMS (Δ = 9.53% vs. 2.82%) and PS_T-CAR_ (Δ = 5.50% vs. 2.10%) than in the CG. Furthermore, changes in VO_2max_ produced higher effect size (d) values for the EG (Δ = 6.67%; d = 0.59) than the CG (Δ = 1.88%; d = 0.18). Both groups improved (p = 0.002) their flying 20-m sprint speed (EG: Δ = 1.01%; CG: Δ = 1.56%). However, small decreases were observed for 10-m sprint speed in the CG (Δ = −2.19%; d = −0.44), while only trivial changes were noticed for the EG (Δ = −0.50%; d = −0.16). Our data support that additional supramaximal ISRT is an effective training stimulus to enhance aerobic performance-related indices and promote small improvements in maximal running speed without impairing the soccer players’ acceleration capacity. This study also shows that PS_T-CAR_ can be useful for individualizing running intensity in supramaximal ISRT modes.

## Introduction

Laboratory and field tests are frequently used at different periods of the season by strength and conditioning coaches as well as practitioners to assess the effectiveness of a specific training program and to monitor the players’ readiness to deal with the demands of training and competition (Svensson and Drust, 2005). Given the stochastic and high-intensity nature of soccer games, aerobic/cardiorespiratory fitness and the ability to perform repeated high-intensity running efforts (soccer-specific fitness) are certainly among the main physical qualities to be developed in soccer players (Mohr et al., 2003; Svensson and Drust, 2005). For instance, a high aerobic fitness level has a fundamental role in soccer for a) playing in top-level soccer leagues (Ziogas et al., 2011); b) covering greater distances at high-speed zones during the game (Fernandes Da Silva et al., 2016; Helgerud et al., 2001; Krolikowska et al., 2023; Rampinini et al., 2007; Rebelo et al., 2014; Ribeiro et al., 2022); and c) enhancing tolerance during intensified training periods (Milanez et al., 2011). Furthermore, aerobically fitter players have shown a lower injury incidence and severity (Gastin et al., 2015), as well as a greater ability to maintain high performance of some key technical actions throughout the game (e.g., kicking speed) than their less fit counterparts (Joseph et al., 2020). This highlights the need to prepare soccer players through well-designed, high-intensity running interventions to support training and match-play demands.

There has been a progressive increase in the relationship between maximal aerobic speed (MAS) (as inferred from V_Vam-Eval_) and the ability to perform high-intensity intermittent running efforts in youth soccer players (U-14: r = 0.67; U-16: r = 0.73; U-18: r = 0.87) (Buchheit and Mendez-Villanueva, 2013). This finding shows that maximal and supramaximal intermittent training strategies should be prioritized during the pubertal and post-pubertal periods to increase or maintain players’ aerobic fitness levels and delay the development of match-induced fatigue (Birat et al., 2018). The applicability, reliability, validity, and sensitivity of several protocols are well known for field-based tests, such as the Yo-Yo Intermittent Recovery Level 1 (Yo-Yo IR1) (Krustrup et al., 2003), 30-15 Intermittent Fitness Test (30-15_IFT_) (Buchheit, 2008), and Carminatti’s Test (T-CAR) (Da Silva et al., 2011; Teixeira et al., 2014) to evaluate the team-sport specific intermittent endurance level. However, the Yo-Yo IR1 is limited in providing accurate data for prescribing training for team sport athletes (Dupont et al., 2010). On the other hand, the 30-15_IFT_ and T-CAR are appealing since both tests allow an individualized training prescription based on their respective end-stage speed (i.e., the so-called ‘peak speed’). While the 30-15_IFT_ is a well-accepted and recognized field test in the literature (Buchheit and Laursen, 2013; Buchheit, 2008; Buchheit et al., 2008; Delextrat et al., 2018), the T-CAR has provided a promising possibility to individualize intermittent shuttle-run training for team sport athletes (Da Silva et al., 2011; Fernandes Da Silva et al., 2015).

Interestingly, prior studies have shown that PS_T-CAR_ did not differ from the speed at VO_2max_ (i.e., MAS), determined by a treadmill incremental running test (Berkowicz et al., 2021;Da Silva et al., 2011; Teixeira et al., 2014) and by incremental track-based tests (Carminatti et al., 2013). This similarity between PS_T-CAR_ and MAS has not been observed for the peak speed derived from other traditional shuttle field tests, such as the Yo-Yo IR Level 1 and the 30-15_IFT_(Buchheit, 2008). For instance, the final speed reached at the 30-15_IFT_ (PS_30-15IFT_) is, on average, 15–25% above MAS (Buchheit, 2008). Although PS_T-CAR_ is probably slower than the PS_30-15IFT_ (hypothesis to be tested in the future), this variable is also highly specific and suitable for programming interval training sessions in team sport athletes, as it also takes into consideration the players’ ability of inter-effort recovery and change of direction ability.

The peak running speed reached at the end of the T-CAR (PS_T-CAR_) is well related to VO_2max_ and MAS (Da Silva et al., 2011; Teixeira et al., 2014), the maximal lactate steady state (Carminatti et al., 2021), and repeated sprint ability (Da Silva et al., 2011) in soccer players. In addition, PS_T-CAR_ has been significantly associated with the distance covered at high-intensity activities during friendly matches (11 vs. 11) and medium-sided games (7 vs. 7) in youth soccer players (Fernandes Da Silva et al., 2016). As practical implications for training prescription, the use of the PS_T-CAR_ as a reference to individualize running intensity is effective in eliciting and sustaining VO_2max_ during an intermittent shuttle running session (100% PS_T-CAR_: 12-s effort : 6-s rest) performed until volitional exhaustion in futsal players (Floriano et al., 2016).

To the best of our knowledge, only one study has investigated the adaptive responses after a training period (5 weeks) using PS_T-CAR_ as the reference speed to individualize the intensity during intermittent shuttle-run training sessions in soccer players (Fernandes Da Silva et al., 2015). In that study, the relative training intensity was set at 100% PS_T-CAR_ in both training models (6-s effort : 6-s rest [straight-line] and 12-s effort :12-s rest [shuttle-run]). Considering that PS_T-CAR_ has been identified as a variable close to MAS (Da Silva et al., 2011; Teixeira et al., 2014), future studies should investigate the accuracy of PS_T-CAR_ to individualize running distances at supramaximal intensities during intermittent shuttle-run exercise protocols since some researchers have questioned the use of MAS to calibrate running distances in aerobic training interventions involving supramaximal intensities (Buchheit and Laursen, 2013). The findings can better inform coaches and practitioners if the use of PS_T-CAR_ as a reference running speed is limited to training intensities close to 100% MAS or it can be used in higher intensities zones (e.g., 115% MAS).

Therefore, the primary purpose of this study was to investigate the effectiveness of a supramaximal shuttle running-training model prescribed from the T-CAR (at 115% PS_T-CAR_) in inducing changes in LMS, VO_2max_, PS_T-CAR_, and sprinting speed in male junior soccer players.

## Methods

### Participants

Twenty-three male soccer players from a Brazilian national-level team (mean ± SD; age 18.07 ± 0.9 y, body height 1.76 ± 0.65 m, body mass 71.9 ± 8.7 kg) volunteered to participate in this study. Athletes had a minimum of three years of soccer-specific training experience and trained six days per week, with one or two daily training sessions. The inclusion criteria were i) regular participation in 85% of training sessions during the period of investigation; and ii) not suffering from injuries during the study period and not taking any medication that could alter the outcomes of this study. Parents, legal guardians, and club managers were informed about the nature of the study, including objectives, protocols, and related risks, and provided informed written consent. Participation was voluntary, and players provided assent after being informed that they could withdraw from the study at any time. This study was conducted following the principles of the Declaration of Helsinki, and approved by the Ethics Committee of the Federal University of Santa Catarina (protocol code 2.047.140, approved on 04 May 2017).

### Measurements

Before each test, soccer players performed a standardized warm-up. A 30-m sprint running test was performed to determine the maximal sprinting speed, where a photocell system (Microgate, Bolzano, Italy) was placed on the starting line at the first 10 m and 30 m. The maximal sprinting speed was defined by the best time of the flying 20-m sprint observed during three attempts.

The T-CAR consisted of intermittent shuttle runs of 12 s between two lines set at progressive distances, with a 6-s recovery period between each run and a total stage time of 90 s. The protocol had a starting average speed of 9 km•h^−1^ over a running distance of 30 m (15 m back and forth). The length in a single direction was increased progressively by 1 m at every stage. Thus, each stage consisted of five repetitions with a 6-s walking period between two lines set 2.5 m from the starting line. Eight to ten athletes were evaluated simultaneously with the running pace dictated by a pre-recorded audio system (Da Silva et al., 2011). The test ended when participants failed to follow the audio cues on the front line for two successive repetitions (objective criteria determined by observers). PS_T-CAR_ was calculated from the distance of the last set completed by the athlete divided by the time to complete the stage repetition. When an incomplete set occurred, peak speed was interpolated using the equation: PS = v + (ns/10)*0.6, where “v” is the speed of the last fully completed stage and “ns” is the number of repetitions completed in the partially completed stage.

The Anaerobic Speed Reserve (ARS) was calculated by the difference between the maximal sprint speed (flying 20-m sprint speed) and the maximal aerobic speed (PS_T-CAR_).

In the laboratory, athletes performed an adapted lactate minimum protocol (Tegtebur et al., 1993), compounded by the Wingate test (i.e., 30-s all-out cycling sprint) and an incremental treadmill test. Thus, the lactate minimum running speed (LMS) was determined using a protocol composed of the Wingate test aiming to induce hyperlactatemia, followed by 8 min of passive rest and an incremental running test until exhaustion. The Wingate test was performed on an electronically braked Lode Excalibur Sport Cycle Ergometer (Lode, Groningen, The Netherlands). The resistance was set as a torque factor (Nm) based upon the participant’s body mass, which was 0.075 Nm/kg. During the Wingate test, participants were instructed to develop and maintain a maximal pedal speed throughout the 30-s period. Capillary blood samples (25 μ l) were obtained at the first and seventh minutes of recovery, and blood lactate concentration ([La]) was assessed using an electrochemical analyzer (YSL 2700 STAT, Yellow Springs, Ohio, USA). The incremental running test was conducted on a motor-driven treadmill (Imbramed Millennium Super, Porto Alegre, Brazil) starting at 10 km·h^−1^ for 3 min, followed by increments of 1 km·h^−1^ every 3 min until volitional exhaustion. The treadmill incline was set at a 1% gradient. Immediately after the completion of each stage, capillary blood samples were taken from the ear lobe to determine [La] concentration. LMS was determined by visual inspection as the lowest lactate value observed during the incremental test (Tegtebur et al., 1993).Respiratory and pulmonary gas exchange variables were measured breath-by-breath during the incremental phase (Quark PFTergo, Cosmed, Rome, Italy), and the data were reduced to 15-s averages. VO_2max_ was defined as the highest value obtained in a 15-s interval. The Lactate Minimum protocol was previously shown to provide valid measures of VO_2max_ compared to an incremental step test (De Lucas et al., 2003). The attainment of VO_2max_ was defined using the criteria proposed by Howley et al. (1995).

### Design and Procedures

**Figure 1:**
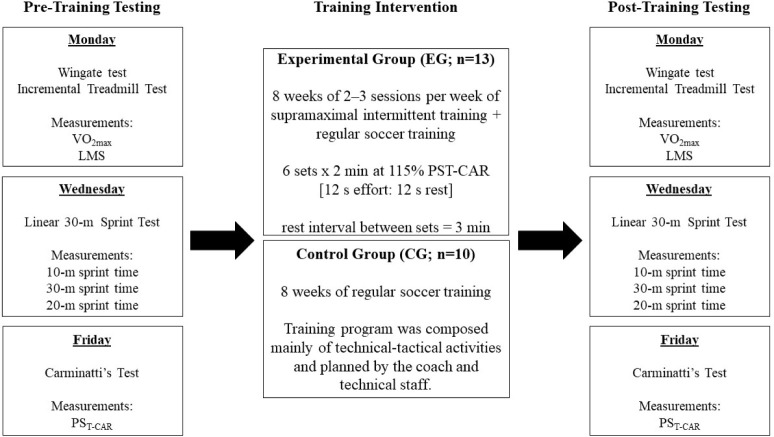
Schematic representation of the study design.

A parallel between two groups, randomized and longitudinal experimental design was used in this study to verify the additional effects of supramaximal running interval training prescribed from PS_T-CAR_ versus traditional regular soccer training on physiological and performance adaptations in male junior soccer players during the preseason phase. Players were randomly assigned to either a control group that comprised normal soccer training sessions (CG; n = 10) or an experimental group (EG; n = 13), with added sessionsof supramaximal (i.e., 115% PS_T-CAR_) intermittent training over eight weeks. Players within each group were equally matched according to their baseline T-CAR performance to ensure that both groups displayed equivalent average pre-training values for the output variables. Performance tests were carried out within seven days before the commencement and after the cessation of the eight-week training intervention period and included i) a 30-m sprint test, ii) an incremental treadmill test, and iii) an incremental intermittent shuttle-running test (i.e., T-CAR). These performance tests were conducted in a laboratory and on a soccer grass field. Players were familiarized with the testing procedures before the data collection, i.e., each participant completed all the test protocols at least once before the study commenced. They were asked to consume their last meal (with no caffeine ingestion) at least three hours before the beginning of the testing session. A schematic representation of the study design is depicted in Figure 1.

### Training Intervention

The training intervention program was implemented during preseason months (March and April) in addition to the regular soccer training sessions planned by coaches and technical staff. During the 8-week training period, players completed 72 training sessions. These training sessions were organized as follows: 20 sessions dedicated to the supramaximal training intervention of our study, 12 sessions for developing strength/power capacities, 40 sessions devoted to soccer-specific technical-tactical drills, and four friendly matches.

The supramaximal intermittent interval training program was prescribed based on PS_T-CAR_. Training regimens were undertaken by athletes three times a week (every Monday, Wednesday, and Friday) during the first four weeks and twice a week (Monday and Wednesday) during the last four weeks of the preseason training period. Later, the weekly training frequency was reduced due to the team's preparation for friendly matches that usually took place on Saturdays. The running intensity used was 115% of PS_T-CAR,_ and the work-to-rest ratio during the supramaximal intermittent training sessions was 1:1. The training protocol consisted of shuttle-run intervals organized in six sets of 2-min bouts with 3 min of rest between the sets. Within each 2-min bout, players repeated 12-s shuttle-run bouts (with one change of direction) and 12 s of rest. After the fourth week of training, running intensity was increased by 3% for all participants to maintain the expected relative training intensity. During supramaximal sessions, CG players continued their ordinary weekly training program as planned by coaches and technical staff, mainly composed of technical-tactical activities. No additional shuttle-run-based intermittent training was allowed in the control group.

All training sessions took place on natural grass and were carefully supervised. No other physical exercise was conducted aside from the one prescribed in the soccer environment. Players maintained their standard lifestyle and food intake during the experimental phase to minimize any potential interference of external variables.

### Statistical Analysis

Descriptive statistics are presented as means and standard deviations (SD). Data distribution and variance equality were assessed through the Shapiro-Wilk and Levene’s tests, respectively. When assumptions were violated, log transformations were computed. A two-way repeated measure ANOVA with one between factor (group: EG vs. CG) and one within factor (time: pre-training vs. post-training) was used for each dependent variable to examine the effects of intervention (EG vs. CG) on aerobic fitness measures and sprinting speed. Bonferroni corrected post hoc tests were performed to determine pairwise differences if a significant F value was identified for group-by-time interactions. The significance level was set at *p* ≤ 0.05. In addition, effect sizes (Cohen’s *d*) were calculated from eta-squared using the ANOVA output. Moreover, within-group effect sizes were computed using the following equation: Effect size = (mean_post − mean_pre)/pooled SD. Threshold values for Cohen’s *d* statistics were 0.20, 0.60, 1.20, 2.0, and 4.0 for small, moderate, large, very large, and extremely large effects, respectively (Hopkins et al., 2009). All statistical analyses were carried out using SPSS (SPSS 17.0 version, Chicago, Illinois, USA).

## Results

### Cardiovascular and Metabolic Responses during Supramaximal Training Sessions

**Figure 2:**
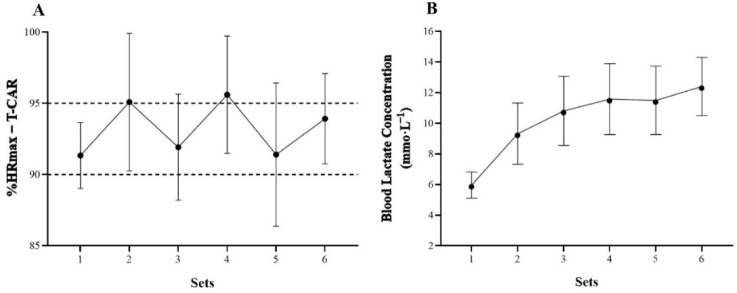
Heart rate (HR) response (panel A) and blood lactate concentration [La] (panel B) over the sets during supramaximal running training performed at 115% PS_T-CAR_.

Mean heart rate (HR) values obtained during a single supramaximal training session at 115% PS_T-CAR_ are depicted in Figure 2. The same Figure illustrates mean [La] concentration assessed immediately after each running set during seven training sessions.

### Baseline

No significant differences between groups were identified for all analyzed measures at baseline (*p*> 0.05) (Table 2).

**Table 1:** Training program implemented during the 8-week intervention.

					Experimental Group (EG)			Control Group (CG)		
Supramaximal Intermittent Training					number of sessions = 20			number of sessions = 0		
					see below			not assigned		
Strength-Power Training					number of sessions = 12			number of sessions = 12		
					Strength-Power training exercises (sets/repetition): Half-Squat (3/8), Hip Thrust (3/8), Lunge (3/8), Bench Press (3/8), Pull-down (3/8), depth jump (3/6), Linear horizontal hurdle jump (3/6) and Linear vertical hurdle jump (3/6)					
Technical-Tactical Training					number of sessions = 40			number of sessions = 40		
					TT training comprised the following activities and types of small-sided games (SSG’s): Passing drills with specific tactical combinations Ball possession drills Games aiming progression into the pitch Transition drills SSG’s 4x4; 5x5; 7x7; 9x9 with goalkeepers Attack x Defense situations with numeric superiority 10 x 8 with goalkeepers; 10 x 10 with goalkeepers in 1/2 pitch size and 10 x 10 with goalkeepers in ¾ pitch size. Set Pieces					
Friendly Matches					number of matches = 4			number of matches = 4		
Total					76			56		
Supramaximal Intermittent Shuttle-Run Training Program Implemented for the Experimental Group										
	Intensity		Repetition*			Sets*			Total Volume per Session	
	PS_T-CAR_ (km•h^−1^)	115% PS_T-CAR_ (km•h^−1^)	Distance (m)		Number (n)	Distance (m)		Number (n)	Distance Covered (m)	
Player 1	15.6	17.9	59.8		5	299.0		6	1794.0	
Player 2	15.8	18.2	60.6		5	302.8		6	1817.0	
Player 3	16.2	18.6	62.1		5	310.5		6	1863.0	
Player 4	16.3	18.7	62.5		5	312.4		6	1874.5	
Player 5	16.5	19.0	63.3		5	316.3		6	1897.5	
Player 6	16.6	19.1	63.6		5	318.2		6	1909.0	
Player 7	16.7	19.2	64.0		5	320.1		6	1920.5	
Player 8	16.9	19.4	64.8		5	323.9		6	1943.5	
Player 9	17.4	20.0	66.7		5	333.5		6	2001.0	
Player 10	17.5	20.1	67.1		5	335.4		6	2012.5	
Player 11	17.9	20.6	68.6		5	343.1		6	2058.5	
Player 12	17.9	20.6	68.6		5	343.1		6	2058.5	
Player 13	18.0	20.7	69.0		5	345.0		6	2070.0	
**Mean**	**16.87**	**19.40**	**64.67**		**5**	**323.33**		**6**	**1939.96**	
**SD**	**0.81**	**0.93**	**3.10**		**0**	**15.50**		**0**	**93.00**	

* Each repetition lasted 12 s of effort interspersed by 12 s of rest; Each set lasted 120 s.

**Table 2:** Comparisons of the physiological indexes and running performance outcomes before and after the training period in both control (CG) and experimental (EG) groups. Values are presented as mean ± SD.

				Repeated measures two-way ANOVA					
		Before Training	After Training	Time Effect		Group Effect		Group-by-Time	
	Training Group	Mean ± SD	Mean ± SD	F	*p*	F	*p*	F	*p*
								
10-m sprint speed (km•h^−1^)	CG (n = 10)	20.49 ± 0.97	20.05 ± 1.01	**4.432**	**0.047**	0.075	0.787	1.487	0.236
	EG (n =13)	20.18 ± 0.64	20.08 ± 0.62						
									
30-m sprint speed (km•h^−1^)	CG (n = 10)	25.69 ± 0.99	25.73 ± 1.07	0.862	0.364	0.043	0.838	0.232	0.635
	EG (n =13)	25.57 ± 0.73	25.67 ± 0.73						
									
Flying 20-m sprint speed (km•h^−1^)	CG (n = 10)	29.53 ± 1.24	29.98 ± 1.08	**13.241**	**0.002**	0.019	0.890	0.593	0.450
	EG (n =13)	29.53 ± 0.91	29.83 ± 1.01						
									
Lactate Minimum Speed (km•h^−1^)	CG (n = 6)	11.83 ± 0.41	12.17 ± 0.41	**17.303**	**0.001**	0.335	0.572	**4.951**	**0.043**
	EG (n =10)	11.60 ± 0.70	12.70 ± 0.67						
									
VO_2max_ (mL•kg^−1^•min^−1^)	CG (n = 10)	54.12 ± 4.84	55.09 ± 4.41	**11.205**	**0.003**	2.455	0.132	3.738	0.067
	EG (n =13)	55.92 ± 5.75	59.55 ± 4.79						
									
PS_T-CAR_ (km•h^−1^)	CG (n = 10)	16.77 ± 0.79	17.12 ± 0.74	**46.154**	**<0.001**	1.447	0.242	**9.352**	**0.006**
	EG (n =13)	16.87 ± 0.81	17.80 ± 0.81						
									
ASR (km•h^−1^)	CG (n = 10)	12.76 ± 1.01	12.86 ± 1.08	3.029	0.096	0.886	0.357	**6.359**	**0.020**
	EG (n =13)	12.66 ± 1.06	12.03 ± 1.48						

Note: bold values indicate statistical significance (*p*< 0.05).

The analysis did not reveal a significant main effect of time nor a significant group-by-time interaction for the 30-m sprint speed (*p*> 0.05; *d* = 0.40 and 0.21). However, a significant main effect of time was observed for 10-m sprint speed (F_(1,21)_ = 4.432; *p* = 0.047; *d* = 0.92), flying 20-m sprint speed (F_(1,21)_ = 13.241; *p* = 0.002; *d* = 1.59) and VO_2max_ (F_(1,21)_ = 11.205; *p* = 0.003; *d* = 1.46). A trend toward a significant group-by-time interaction was observed for VO_2max_ (F_(1,21)_ = 3.738; *p* = 0.067; *d* = 0.84). Changes in VO_2max_ produced ES values larger for the EG (Δ = 6.67%; *d* = 0.59) than the CG (Δ = 1.88%; *d* = 0.18). There was a significant group-by-time interaction for LMS (F_(1,14)_ = 4.951; *p* = 0.043; *d* = 1.19), PS_T-CAR_ (F_(1,21)_ = 9.352; *p* = 0.006; *d* = 1.33) and ASR (F_(1,21)_ = 6.359; *p* = 0.020; *d* = 1.10). Post-hoc tests revealed that pre vs. post improvements in LMS (Δ = 9.53% [*p*< 0.001; *d* = 1.44] vs. 2.82% [*p* = 0.241; *d* = 0.69]), PS_T-CAR_ (Δ = 5.50% [*p*< 0.001; *d* = 1.07] vs. 2.10% [*p* = 0.021; *d* = 0.40]) and ASR (Δ = −5.32% [*p* = 0.004; *d* = −0.55] vs. 0.84% [*p* = 0.609; *d* = 0.10]) were larger for the EG compared to the CG.

## Discussion

**Figure 3:**
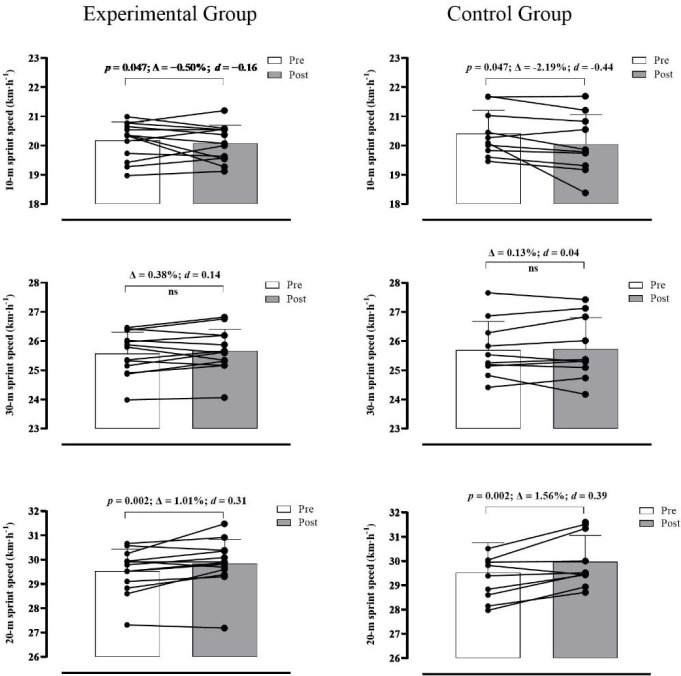
Training-induced changes (mean ± SD; with individual values; relative change (Δ) and *d* values) for 10-m, 30-m and flying 20-m sprint speed measures in the experimental (left panels) and control (right panels) groups. Note: ns—not significant

**Figure 4:**
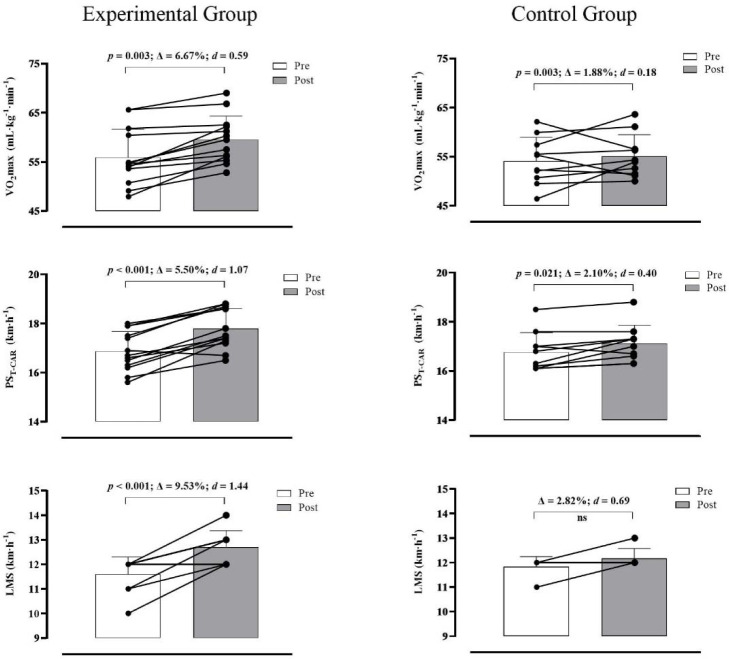
Training-induced changes (mean ± SD; with individual values; relative change (Δ) and *d* values) for VO_2max_, peak speed reached at the end of the Carminatti’s Test (PS_T-CAR_) and lactate minimum speed (LMS) variables in the experimental (left panels) and control (right panels) groups. Note: ns—not significant

The present study aimed to examine whether adding supramaximal running-based exercises prescribed from the T-CAR would induce superior gains in aerobic variables (i.e., PS_T-CAR_, VO_2max_, and LMS) and sprinting speed in comparison with the traditional regular soccer training during the preseason. The main findings of this study confirmed our initial hypothesis partly. Replacing some technical-tactical soccer drills with supramaximal running-based exercises prescribed from PS_T-CAR_ was an efficient training strategy to induce larger adaptations in key aerobic fitness indices (Figure 4). Furthermore, eight weeks of training based on supramaximal intensity (i.e., 115% PS_T-CAR_) were also capable of maintaining 30-m sprint time while improving flying 20-m sprint times (moderate to large ES). However, trivial and small within-group decreases in 10-m sprint speed were noticed in EG (*d* = 0.16) and CG (*d* = 0.44) groups (Figure 3).

It is worth noting that the main outcome of the T-CAR (i.e., PS_T-CAR_) allows individualizing the running speed during training while several athletes perform the same session.In this way, this test differs from other shuttle-run-based tests (e.g., Yo-Yo IR Level 1) and provides a possibility to prescribe sub-maximal, maximal, and supramaximal shuttle-run training. In a previous study, Fernandes da Silva et al. (2015) compared the effects of two interval training models prescribed from PS_T-CAR_ on aerobic running performance in soccer players. However, an important limitation of that study was the lack of a control group, as mentioned by the authors, especially because it was impossible to ensure that interval training regimes only elicited positive changes in aerobic fitness variables. The present study included a control group with players maintaining their traditional regular soccer training. Our findings add further information to the literature showing the efficiency of a supramaximal interval training program prescribed from PS_T-CAR_ in inducing larger enhancements in key aerobic fitness variables during the preseason.

As expected, the experimental training protocol outlined in our study elicited high cardiovascular and metabolic stresses on players (Figure 2). The mean heart rate (HR) values throughout the six sets of the training protocol ranged from 91% to 95% of the individual maximal HR. These values fall within the optimal training intensity zone assumed to elicit a high cardiovascular load and result in an optimized physical performance (Buchheit and Laursen, 2013; Castagna et al., 2011; Helgerud et al., 2001). There was also a progressive increase in [La] concentration over the six sets of the training protocol. Players began training with [La] concentration of between 5 and 6 mmol•L^−1^ and completed the session accumulating [La] concentration of between 12 and 13 mmol•L^−1^. According to the classification proposed by Buchheit and Laursen(2013), [La] concentration in our training protocol ranged from moderate (> 6 mmol•L^−1^) to very high (≥ 14 mmol•L^−1^) values. These values highlight the high glycolytic energy contribution during supramaximal running-based training based on the T-CAR.

In this study, small decreases (Δ = −2.2%; *d* = −0.44) in 10-m sprint speed were noticed for the CG, but only trivial changes for the EG. This finding observed in the CG is similar to that reported by Nakamura et al. (2016) whose results also showed substantial reductions (*d* = −0.51 and −0.43) in acceleration capacity (i.e., 5-m and 10-m sprint speed) of under-20 professional male futsal players. A possible explanation for this acceleration impairment over shorter distances may be the low volume of horizontally-directed exercises (e.g., horizontal jumps, resisted sprints and shuttle-run drills) performed by athletes throughout the preseason phase. Given that our supramaximal training involved execution of several shuttle-runs, the possibility that this increased time spent accelerating may have contributed to the lower decrease of 10-m sprint speed observed in the EG should not be disregarded. For instance, intermittent shuttle-run training models (due to their directional changes) have been considered effective strategies to maintain or enhance the speed-power-related abilities in team sport players (Campos et al., 2021; Teixeira et al., 2018). Another reason for the decrease in 10-m sprint speed following the intervention period could be related to the high-volume of technical-tactical drills (i.e., high endurance demand) and their potential concurrent effects on sprinting ability during a short-term period of the preseason phase, which is also featured by high training loads and accumulated fatigue (Nakamura et al., 2016).

The ability to perform multiple high-intensity intermittent efforts combined with short recovery periods has been a physical quality considered crucial to soccer performance (Buchheit and Mendez-Villanueva, 2013; Fernandes Da Silva et al., 2016; Helgerud et al., 2001; Rebelo et al., 2014). In this study, we showed significant (moderate) improvements in PS_T-CAR_ after eight weeks of interval training at 115% PS_T-CAR_ (EG), while only small changes in PS_T-CAR_ were observed in those who performed traditional preseason soccer training (CG). Initially, larger increases in PS_T-CAR_ in the EG may be partially explained by the nature of the training protocol, as supramaximal shuttle-run-based interval training was based on the T-CAR. In this regard, it is reasonable to assume that positive changes in the specific coordination of the lower limbs to change direction may have occurred due to the involvement of the same muscles during the several shuttle-run actions executed in the interval training session. The change in PS_T-CAR_ was not correlated with gains in VO_2max_ (an indicator of central adaptation to training) in the EG of this study. On the other hand, in a recent study using youth basketball players, Delextrat et al. (2018) showed that improvements in the final speed of 30-15_IFT_ (comparable with the T-CAR used here) were related to better muscle reoxygenation after maximal running sprints. It highlights that a faster muscle reoxygenation rate in the recovery after high-intensity exercises probably indicates a better muscle aerobic function. It could be an important peripheral adaptation supporting the improvements in the ability to perform repeated high-intensity intermittent efforts.

The magnitude of change in PS_T-CAR_ for the EG in our study is comparable to those reported in other experimental studies examining the effectiveness of different interval training regimes (Buchheit et al., 2008; Fernandes Da Silva et al., 2015; Ferrari Bravo et al., 2008). Previous studies have consistently shown that PS_T-CAR_ is a reliable and useful variable to estimate MAS in soccer and futsal players under field conditions (Da Silva et al., 2011; Dittrich et al., 2011; Teixeira et al., 2014). In practical terms, players from both groups enhanced their maximal aerobic power. However, the increases in PS_T-CAR_ were more significant in the EG (Figure 4), probably resulting in a greater ability to repeat and sustain intermittent running efforts performed above MAS throughout a soccer match. It has been demonstrated that adolescent soccer players with better performance on the T-CAR could perform a greater amount of high-intensity activities during friendly matches, regardless of age, maturity, and body size-associated variations (Fernandes Da Silva et al., 2016). Furthermore, given that small improvements in MSS (flying 20-m sprint speed) after the preseason phase were similar between the groups (Figure 3), superior gains in PS_T-CAR_ in the EG also resulted in a decrease in the anaerobic speed reserve (ASR). From a physiological perspective, it means that these fitter aerobically players probably will use a smaller proportion of their anaerobic work capacity at a given submaximal and supramaximal intensity and, in turn, less reliance on the anaerobic process to supply energy, increasing the players’ exercise tolerance (Buchheit and Laursen, 2013). From a game perspective, greater exercise tolerance can translate into a greater ability to perform and repeat multiple high-intensity running efforts during soccer matches (Buchheit and Laursen, 2013).

Our finding also indicated that the improvement in LMS was significantly (*p* = 0.043) greater in the EG (Δ = 9.53%; *d* = 1.44) than in the CG (Δ = 2.82%; *d* = 0.69) following the intervention period (Figure 4), while a trend (*p* = 0.067 for group-by-time interaction effect) toward superior increases in VO_2max_ was displayed in the EG (Δ = 6.67%; *d* = 0.59) compared to the CG (Δ = 1.88%; *d* = 0.18). Indeed, such adaptations in submaximal and maximal aerobic indices have been positively related to time spent at training intensities > 90% HR_peak_(Castagna et al., 2011). Thus, the larger improvements displayed in the EG than the CG could be attributed to the better adequacy of more controlled shuttle-run-based interval training sessions to reach this training target zone (>90% HR_peak_) and guarantee a similar training stimulus among players than small-sided games or technical-tactical activities. It is well known that interindividual variability in training responses is substantially increased in these game-based activities, indicating greater heterogeneity among players performing the same training session (Massamba et al., 2021). The improvements for the EG of this study were similar to those reported in elite professional Greek soccer players after preseason training (Kalapotharakos et al., 2011). In that study, the training content involved plyometric and speed training strategies, different from those performed by the control group in the current study, which was primarily based on technical-tactical workouts. In another study, Helgerud et al. (2001) found an increase in VO_2max_ in the experimental group after eight weeks of interval training, while no further change was found in the CG. It suggests that traditional preseason soccer training can only provide an insufficient training stimulus to increase LMS and VO_2max_ in well-trained soccer players during the preseason.

In opposition to our findings, Fernandes da Silva et al. (2015) showed no substantial gains in VO_2max_ after five weeks of interval training performed at 100% PS_T-CAR_. McGinley and Bishop (2016) also showed that VO_2max_ did not change after four weeks of two different interval training models. It should be highlighted that the lower training volume of the latter two studies, caused by the short duration of the training program (< 4–6 weeks), might be viewed as a limiting factor to the adaptive response of VO_2max_ in well-trained team-sport athletes. For example, in the current study, players performed a total of 20 training sessions, whereas, in the previous studies, athletes completed about 10–12 training sessions (Fernandes Da Silva et al., 2015; McGinley and Bishop, 2016). Considering that U-19 squads are one of the last competitive categories before reaching the professional level, it is important to implement long-term and well-designed training programs in these ages to support professional soccer demand. Yet, although VO_2max_ has not been a physiological variable linked directly to match running performance (Krustrup et al., 2003; Rebelo et al., 2014), high general and soccer-specific aerobic fitness should be prioritized, aiming to increase the players’ ability to deal with the high workloads placed in intensified training periods, such as the preseason period (Milanez et al., 2011; Miloski et al., 2014).

Some limitations of the current study should be acknowledged. The main limitations were the small sample size and the lack of control of match and training loads during the preseason. Nevertheless, it is worth mentioning that previous studies focusing on the same training intervention have used similar sample sizes (Buchheit et al., 2008; Fernandes Da Silva et al., 2015; Ferrari Bravo et al., 2008). Finally, the absence of match and training load data did not allow us to verify whether both groups (experimental vs. control) accumulated the same internal (heart rate or rating of perceived exertion-based metrics) and external workloads (e.g., global positioning system-based metrics such as total distance and high-speed, sprinting, acceleration and deceleration distances) during the preseason. However, training-induced adaptations observed during the intervention period corroborate the assumption that the higher gains in performance can be attributed to the additional conditioning program (based on PS_T-CAR_) and not to soccer training alone.

## Conclusions

Our results demonstrate the potential of supramaximal running-based training prescribed using PS_T-CAR_ as a variable to individualize running distance (i.e., training intensity). This led to superior gains in performing repeated high-intensity intermittent efforts (i.e., PS_T-CAR_), LMS, and VO_2max_ in junior male soccer players compared to traditional preseason soccer training (control group). This training protocol could help coaches and physical conditioning professionals vary the training stimulus and develop their supramaximal training sessions to prepare team sport players better to ensure the repeated high-intensity actions commonly required during a match.
